# Dysglycemia screening with oral glucose tolerance test in adolescents with polycystic ovary syndrome and relationship with obesity

**DOI:** 10.1186/s12902-022-01098-0

**Published:** 2022-07-16

**Authors:** Jyotsna Gupta, Zoltan Antal, Elizabeth Mauer, Linda M. Gerber, Anjile An, Marisa Censani

**Affiliations:** 1 Department of Pediatrics, Division of Pediatric Endocrinology, New York Presbyterian Hospital, Weill Cornell Medicine, 505 East 70th Street, New York, NY USA; 2 Department of Population Health Sciences, Division of Biostatistics, Weill Cornell Medicine, 1300 York Avenue, New York, NY USA

**Keywords:** PCOS, Obesity, Dysglycemia, BMI, Adolescent

## Abstract

**Background:**

Adolescents with polycystic ovary syndrome (PCOS) are at increased risk of impaired glucose tolerance (IGT) and type 2 diabetes mellitus. The aim of this study is to evaluate dysglycemia and biochemical differences based on BMI status and assess the prognostic ability of elevated hemoglobin A1c (HbA1c) in predicting an abnormal 2 hour oral glucose tolerance test (OGTT).

**Methods:**

Retrospective cohort of female patients aged 11-18 years who underwent 75-g OGTT and were evaluated for PCOS at an urban tertiary care hospital between January 2002 to December 2017.

**Results:**

In 106 adolescents with PCOS who had OGTT results available, IGT was markedly pronounced in the ≥95th percentile BMI group (17 out of 72; 23.6%) compared with <95th percentile BMI group (4 out of 34; 11.7%). One patient with obesity met the criteria for type 2 diabetes. Patients with obesity had significantly higher homeostasis model assessment (HOMA-IR) and lower whole body insulin sensitivity index (WBISI) (*p* < 0.001) compared to patients without obesity. Free testosterone levels were also higher in patients with obesity (*p*< 0.03) and were significantly associated with HOMA-IR when controlling for body mass index (BMI). HbA1c did not demonstrate a strong ability to predict abnormal OGTT on receiver operating characteristic (ROC) curve analysis [Area under the curve (AUC) = 0.572, 95% CI: 0.428, 0.939]).

**Conclusions:**

In a study to assess glucose abnormalities in adolescents with PCOS, IGT was found to be markedly increased in patients with obesity, with abnormal glucose metabolism identified in over one-fifth of the patients. HbA1c alone may be a poor test to assess IGT and we recommend that adolescents diagnosed with PCOS and obesity undergo formal oral glucose tolerance testing.

## Background

Polycystic ovary syndrome is one of the most common endocrine disorders affecting adolescent females [1–4] and is associated with an increased risk of impaired glucose tolerance (IGT), impaired fasting glucose, and type 2 diabetes mellitus. Insulin resistance at least in part contributes to IGT and increased risk of developing type 2 diabetes mellitus [5–7]. The pathophysiology of PCOS involves a combination of factors such as adrenal and ovarian hyperandrogenism, adiposity, insulin resistance and gonadotropin secretion abnormalities [7]. There is a limited body of literature that describes the prevalence of abnormal glucose levels in adolescents with PCOS, and its relationship with obesity is unclear.

Most studies of abnormal glucose tolerance in adolescents with PCOS have a relatively small number of patients and lack a comprehensive evaluation of the associated metabolic abnormalities. In a previous study [5], a group of 66 adolescents with PCOS were analyzed, and IGT was the most common metabolic abnormality identified, occurring with equal frequency in adolescents with and without obesity. In this study, no differences were observed in two-hour insulin, high-density lipoprotein, and testosterone levels in adolescents with IGT despite the marked difference in BMI. This study supported performing an OGTT for adolescents diagnosed with PCOS regardless of BMI, although it was limited by its relatively small sample size and the lack of diversity of its patient population.

Obesity appears to influence metabolic as well as clinical characteristics in patients with PCOS [8, 9]. It is important that obesity be considered in the classification of PCOS phenotypes in order to provide a focus for treatment [10]. There is considerable debate surrounding the optimal screening test for the presence of altered glucose metabolism in this age group. In another study [11] of 163 adolescents, the authors described the prevalence of abnormalities in the glucose levels of adolescent patients with PCOS and supported the use of OGTT as a superior diagnostic test for assessing abnormal glucose levels in adolescents with obesity or overweight status. However, the authors reported limited utility of OGTT in adolescents with PCOS having normal weight. In another study by Gooding et al. [12] consisting of 68 adolescents, more patients were identified to have dysglycemia by HbA1c as compared to OGTT. A study conducted [13] on adult Austrian women with PCOS with median BMI in the normal weight range did not support the use of fasting glucose or HbA1c for the screening of prediabetes in women with PCOS.

In addition, abnormal glucose metabolism in PCOS is associated with many of the known risk factors for metabolic syndrome [14, 15], which needs close examination. Another [16] demonstrated that moderately elevated testosterone concentrations, together with obesity-related inflammatory factors, modify glucose homeostasis by increasing insulin resistance and early insulin secretion.

OGTT is cumbersome and not performed at all centers. In this study, we assessed serum HbA1c levels, which are simpler to perform, for their utility as an indicator of abnormal glucose tolerance in adolescent patients with PCOS. In addition, we also analyzed the relationship between insulin sensitivity and testosterone levels in adolescents with PCOS. It is important to identify which subgroup of patients in this population would benefit most from dysglycemia screening.

## Methods

This study aimed at evaluating dysglycemia and biochemical differences based on BMI status in adolescent female patients and assessing the prognostic ability of elevated hemoglobin A1c (HbA1c) in predicting an abnormal 2-hour oral glucose tolerance test (OGTT).

### Subjects

**Fig. 1:**
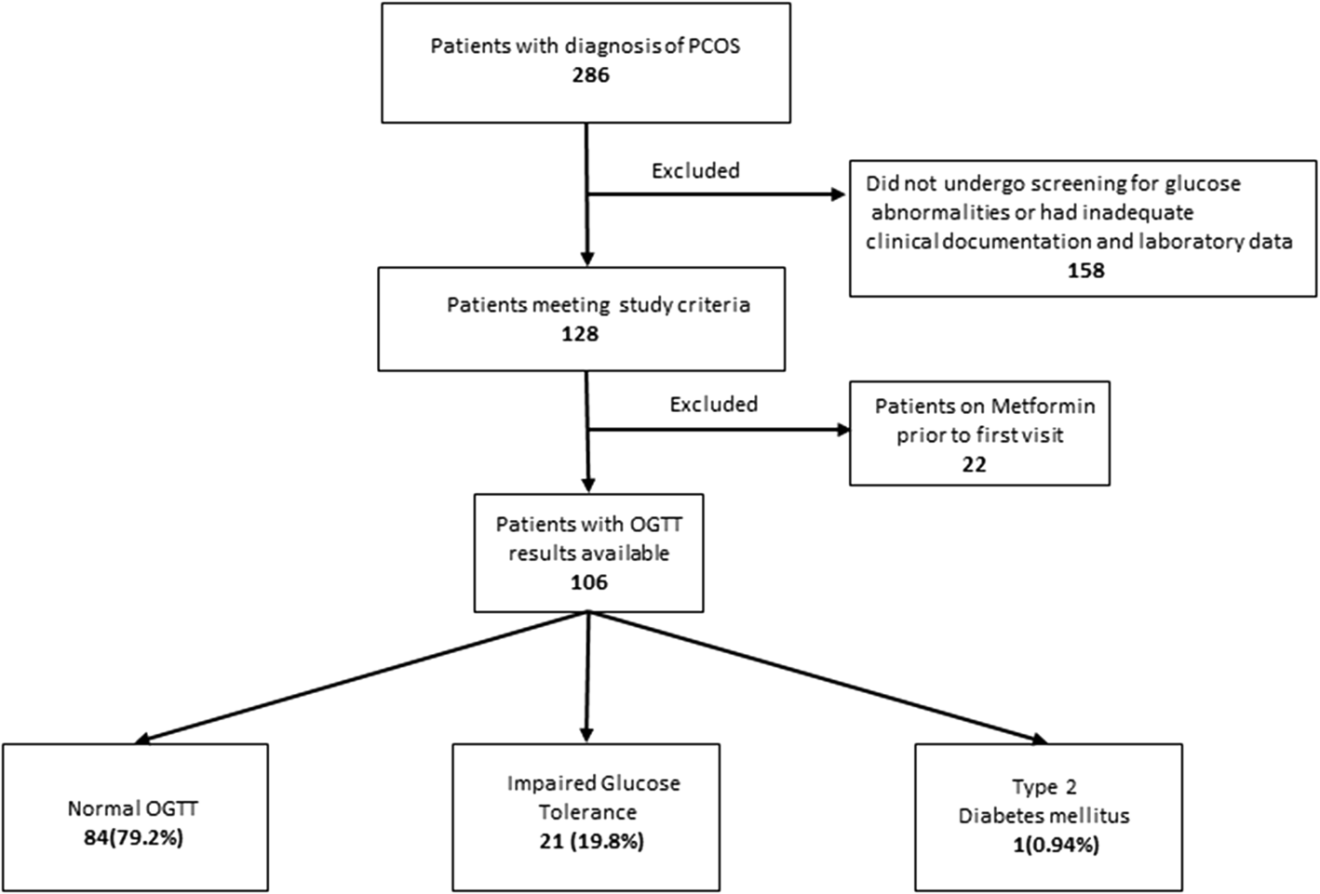
Flowchart indicating study subjects

This retrospective study included female patients with a diagnosis of PCOS aged 11 to 18 years, who were evaluated at the pediatric endocrinology clinics of a tertiary urban academic medical center between January 2002 and December 2017. In order to be included in the study, patients had to have undergone an OGTT and/or HbA1c screening (based on physician preference) as part of routine clinical care. For patients who underwent both OGTT and HbA1c screening, both tests had to be performed within within a three-month time frame [12, 17]. Of the 286 patients identified as having PCOS, 128 patients met the initial inclusion criteria and had the required clinical documentation and laboratory data available. Patients with anemia or other hemoglobinopathies known to affect HbA1c levels were excluded. An additional 22 patients were excluded since they were being treated with metformin, which may affect HbA1c and OGTT results, at the time of initial visit or at the time of lab draw. Ultimately, 106 patients (Fig 1) who underwent oral glucose tolerance testing were included in the final analysis. The collected data included age, ethnicity, weight, BMI, and systolic and diastolic blood pressure.

### Definitions

PCOS was defined as per the Androgen Excess Society (AES) 2009 criteria which includes presence of hyperandrogenism (clinical and/or biochemical), ovarian dysfunction (oligo-anovulation and/or polycystic ovaries), and the exclusion of related disorders [1]. The American Diabetes Association (ADA) criteria were used to classify participants as having normal glucose tolerance (< 140mg/dl), IGT (140–199 mg/dl), and type 2 diabetes (≥ 200mg/dl) at two hours on OGTT [18]. Obesity was defined as having a BMI ≥ 95th percentile for age. The homeostasis model assessment (HOMA-IR) and whole-body insulin sensitivity index (WBISI) calculations were performed to assess insulin resistance [19, 20]. HOMA-IR was calculated using the following formula: HOMA-IR = [fasting insulin (μU/mL) × fasting glucose (mmol/L)] / 22.5, and the normal level was defined as < 3.02 [21]. WBISI was calculated according to the formula by Matsuda et al. [WBISI = 10,000/√(fasting glucose × fasting insulin) × (mean glucose × mean insulin)] with a level < 4.3 predictive of insulin resistance [21, 22].

### Biochemical Assays

Laboratory data extracted from the medical record included HbA1c in addition to 0, 30, 60, 90, and 120-minute glucose and insulin values obtained during the OGTT. Serum glucose measurements were performed using ADVIA® Chemistry XPT systems, and serum insulin measurements were performed using ADVIA® Centaur Insulin assay, a two-site sandwich immunoassay that uses direct chemiluminescent technology. The HbA1c analysis was performed using Tosoh Automated Glycohemoglobin Analyzer, which uses non-porous ion-exchange, high-performance liquid chromatography (HPLC). The additional laboratory data extracted included total and free testosterone, obtained at a certified laboratory (Quest or Labcorp). Free testosterone was obtained by equilibrium dialysis, and total testosterone was obtained using chromatography-mass spectrometry.

### Statistical Analysis

Data were described as N (%) for categorical variables and median [IQR] for continuous variables after assessing for normality with the help of the Shapiro-Wilk tests. Dysglycemia and biochemical differences were compared between BMI groups by Chi-squared/Fisher’s Exact tests or Wilcoxon rank-sum tests. The HOMA-IR and WBISI ratios were calculated. A receiver operating characteristic (ROC) curve was generated to assess the prognostic ability of HbA1c with impaired two -hour glucose tolerance. The relationships between testosterone with HOMA-IR and WBISI were assessed using linear regressions. The study was approved by the Institutional Review Board at Weill Cornell Medical College. All analyses were two-sided, with statistical significance evaluated at the 0.05 alpha level. The analyses were performed in R version 3.5.3 (Vienna, Austria).

## Results

The demographic and biochemical characteristics of our study subjects are detailed in Table 1. The median age of our patients was 15.8 (14.2–16.4) years. Their race/ethnicity was recorded where reported, with the majority of patients (besides other/unknown) reporting Caucasian (25.5%) or Hispanic (16.0%) ethnicity and a fair representation of other race/ethnicities as noted (Table 1). An abnormal two-hour OGTT was found in over one-fifth (21.6%) of our total patient population (Fig. 1).

**Table 1:** Clinical and biochemical characteristics of the study population

	All Patients (*n* = 106)
	N (%) or Median [IQR]
Age (years)	15.8 [14.2–16.4]
Race/Ethnicity	
Asian	8 (7.5%)
Caucasian	27 (25.5%)
African American	11 (10.4%)
Hispanic	17 (16.0%)
Other/Unknown	43 (40.6%)
BMI (kg/m^2^)	30.7 [27.4–35.7]
BMI Z Score	1.96 [1.47–2.25]
BMI Percentile	97.6 [92.7–98.8]
Fasting Glucose (mg/dl)	82.5 [78.0–88.8]
2 hour Glucose (mg/dl)	108.0 [91.2–132]
HbA1c % (n = 89)	5.40 [5.20–5.80]

*IQR* Interquartile range, *BMI* Body Mass Index, *HbA1c* Hemoglobin A1c

**Fig. 2:**
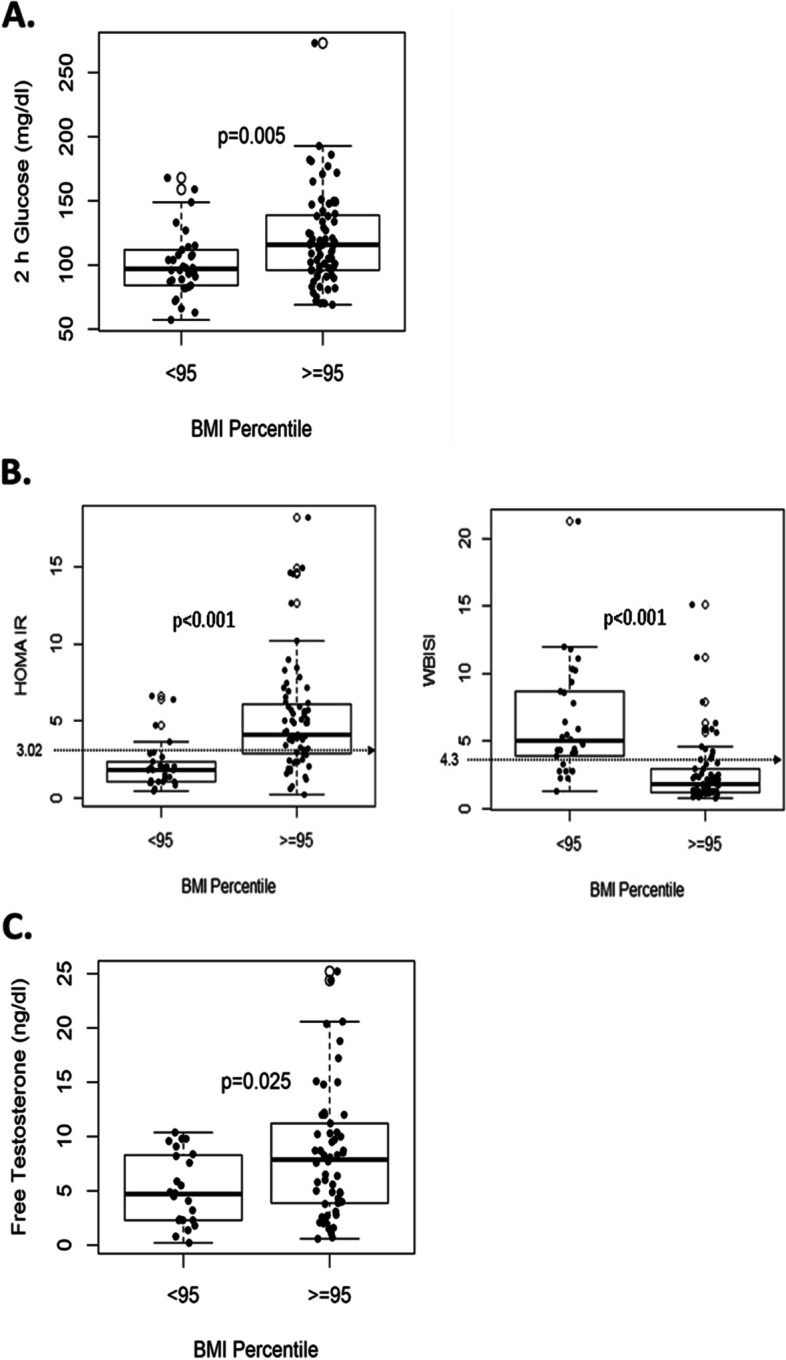
Differences in biochemical characteristics between the < 95th percentile and ≥ 95th percentile groups for **A** 2 h glucose, **B** HOMA-IR and WBISI, and **C** free testosterone levels

The median BMI was 30.7 (interquartile range 27.4–35.7) kg/m2, and the median BMI z-score was 1.96 (interquartile range 1.47–2.25). Significant differences in several biochemical characteristics were observed between patients with obesity (*N*=72) in comparison with those without obesity (*N*=34; Table 2). A statistically significant difference between the two-hour glucose levels during the OGTT between patients without obesity [median 97 mg/dl (range 84.8–111)] and those with obesity [median 116 mg/dl (96.0–138.0)] (*p* = 0.005) (Fig. 2A) was also observed. Impaired glucose tolerance occurred more frequently in subjects in the >95th percentile BMI group (17 out of 72, 23.6% when compared to those in the <95th percentile BMI group (4 out of 34, 11.7%) ) (Table 2). One patient with obesity met the criteria for type 2 diabetes (two-hour glucose of 273 mg/dl and HbA1c 8.1%).

**Table 2:** Biochemical characteristics of adolescent PCOS subjects stratified by BMI

	BMI < 95th percentile (***N*** = 34) Median [IQR]	BMI **>** 95th percentile (***N*** = 72) Median [IQR]	***P***
**Age at time of OGTT**	16.1 [15.6–16.8]	15.2 [13.3–16.1]	0.002**
**BMI (kg/m**^**2**^**)**	25.4 [23.5–27.6]	34.7 [30.6–38.1]	< 0.001***
**BMI z-score**	1.15 [0.87–1.43]	2.17 [1.95–2.38]	< 0.001***
**HbA1c level (%) (N = 89)**	5.40 [5.10–5.53]	5.50 [5.20–5.80]	0.147
**Results of OGTT**			0.325
Normal	30 (88.2%)	54 (75.0%)	
Impaired Glucose Tolerance	4 (11.7%)	17 (23.6%)	
Type 2 Diabetes Mellitus	0 (0.00%)	1 (1.4%)	
**2-hour glucose level (mg/dl)**	97.0 [84.8–111.0]	116 [96.0–138.0]	0.005**
**HOMA-IR (N = 103)**	1.79 [1.04–2.36]	4.02 [2.82–5.94]	< 0.001***
**WBISI (N = 103)**	5.13 [3.90–8.64]	2.00 [1.23–3.21]	< 0.001***
**Total testosterone (ng/dl) (N = 102)**	44.0 [26.5–53.5]	45.0 [37.0–56.0]	0.293
**Free testosterone (ng/dl) (N = 78)**	4.70 [2.30–8.25]	7.90 [3.92–11.0]	0.026*
**LH/FSH ratio (N = 94)**	2.19 [0.94–2.68]	1.54 [1.00–2.30]	0.328
**LH/FSH diagnostic (N = 94)**			0.028*
<= 2	13 (43%)	43 (67%)	
> 2	17 (57%)	21 (33%)	

*IQR* Interquartile range, *OGTT* Oral Glucose Tolerance Test, *BMI* Body Mass Index, *HbA1c* Hemoglobin A1c, *HOMA-IR* Homeostasis Model Assessment, *WBISI* Whole Body Insulin Sensitivity Index

***P ≤ 0.001, **P ≤ 0.01, *P ≤ 0.05

Patients with PCOS and obesity had significantly higher insulin resistance on OGTT as defined by HOMA-IR (4.02 vs 1.79) and lower insulin sensitivity as defined by WBISI (2.00 vs 5.13) compared to the group without obesity (p < 0.001 for both HOMA-IR and WBISI) (Fig. 2B). The HbA1c level was not statistically different in subjects with obesity compared to those without obesity, with median levels in the normal range (<5.7%) for both groups (Table 2). Eighty percent of the patients without obesity had normal HbA1c values as compared with 61.5% in patients with obesity. Those with obesity also had significantly higher free testosterone levels compared to patients without obesity [7.90 ng/dl (interquartile range 3.92–11) vs. 4.70 ng/dL (interquartile range 2.30–8.25), p = 0.025] (Fig. 2C).

**Fig. 3:**
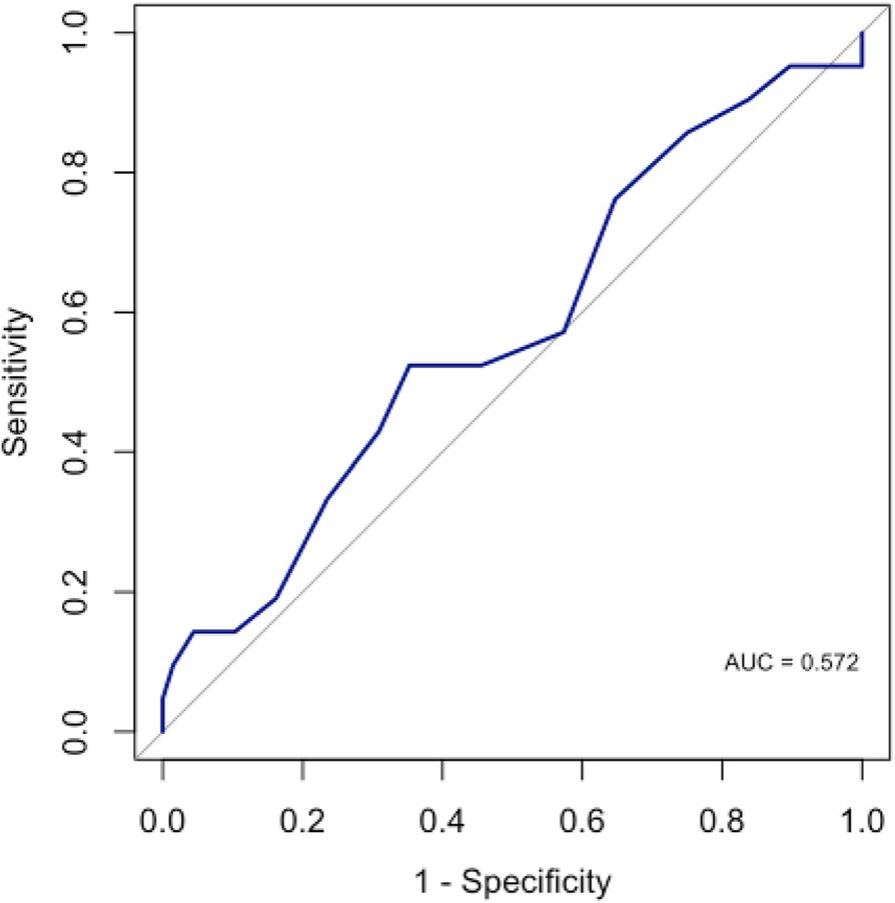
ROC curve of HbA1c predicting abnormal OGTT

Of all study patients, 89 (out of 106) had both OGTT and HbA1c results available within a three-month period. The median HbA1c for the population was 5.40% (interquartile range 5.20–5.80) (Table 1). There was no significant difference in the proportion of patients with IGT between normal and abnormal HbA1c groups (20.3% - 12/59 vs. 30% - 9/30, respectively; p=0.310). HbA1c did not demonstrate a strong ability to predict abnormal 2 hour OGTT and the ROC analysis suggested that HbA1c alone may be a poor predictor of two-hour IGT (AUC = 0.572, 95% CI: 0.428, 0.939) (Fig. 3).

Using a linear regression analysis for patients with available testosterone levels, we found that the total serum testosterone level was significantly associated with HOMA-IR alone (β = 0.031, 95% CI: 0.005-0.06) and approached statistical significance with HOMA-IR when controlling for BMI (β = 0.022, 95% CI: -0.001-0.045). Free testosterone was significantly associated with HOMA-IR alone (β = 0.214, 95% CI: 0.091-0.336) and also when controlling for BMI (β = 0.151, 95% CI: 0.033-0.269). Neither total testosterone nor free testosterone were significantly associated with WBISI, either alone or when controlling for BMI (Table 3).

**Table 3:** Linear regression of testosterone in relation to HOMA-IR and WBISI

**HOMA-IR**				
	**Univariate**		**Controlling for BMI**	
	**estimate (95% CI)**	***P***	**estimate (95% CI)**	***P***
**Total testosterone (ng/dl)**	0.031 (0.005, 0.06)	0.024*	0.022 (–0.001, 0.045)	0.069
**Free testosterone (ng/dl)**	0.214 (0.091, 0.336)	0.001**	0.151 (0.033, 0.269)	0.014*
**WBISI**				
	**Univariate**		**Controlling for BMI**	
	**estimate (95% CI)**	***P***	**estimate (95% CI)**	***P***
**Total testosterone (ng/dl)**	–0.026 (–0.054, 0.003)	0.080	–0.017 (–0.043, 0.009)	0.203
**Free testosterone (ng/dl)**	–0.110 (–0.248, 0.027)	0.119	–0.033 (–0.162, 0.096)	0.620

*BMI* Body Mass Index, *HOMA-IR* Homeostasis Model Assessment, *WBISI* Whole Body Insulin Sensitivity Index

***P ≤ 0.001, **P ≤ 0.01, *P ≤ 0.05

## Discussion

Our study describes dysglycemia and its association with biochemical characteristics in adolescent girls with PCOS. Dysglycemia has been implicated as an independent risk factor in the development of type 2 diabetes mellitus as well as cardiovascular diseases [22, 23], indicating the need for its screening in all at risk populations, including adolescents with PCOS who may not have clinically apparent symptoms of abnormal glucose metabolism. Over one-fifth of our adolescent cohort was found to have IGT. Since we included only patients who had undergone a formal OGTT test, the true prevalence of these glucose abnormalities may be higher in this population. Our findings are in line with others who have noted similar rates of abnormal glucose metabolism in adolescents with PCOS [5, 11].

We additionally found that although the glucose abnormalities can occur in both obese and non-obese BMI ranges, an increased BMI of ≥ 95th percentile places patients at a higher risk of dysglycemia in this patient group, with over one-fourth of these patients meeting the criteria for IGT. In line with the findings of a previous study [5], we found that IGT was the most common glucose abnormality on the OGTT, emphasizing the importance of lifestyle changes and maintaining a healthy BMI in patients with PCOS. We additionally found that patients with obesity had greater insulin resistance and lower insulin sensitivity based on HOMA-IR and WBISI indices respectively, and these differences were clinically significant. To our knowledge, previous studies in adolescent patients with PCOS have not described these associations in detail. Our study emphasizes that the calculation of these indices may provide more insight into the glucose metabolism abnormalities in this patient population.

There has been a debate on the optimal screening test for diagnosing glucose abnormalities in adolescents with PCOS, and the recommendations have been varied. A study [11] showed the limited utility of dysglycemia screening using OGTT in adolescents with normal weight (BMI < 85th percentile) having PCOS. In 2007, the Androgen Excess Society recommended that all adult patients with PCOS be screened for IGT with a two-hour OGTT and a few members of the AES board recommended alternatively screening women with PCOS for IGT and type 2 diabetes using an OGTT only in patients with additional risk factors [22]. The American Diabetes Association stated in the recent 2019 standard of medical care in diabetes that testing for prediabetes should be considered in adults with obesity or overweight (BMI ≥ 25 kg/m^2^ or ≥ 23 kg/m^2^ in Asian Americans) diagnosed with PCOS. These recommendations were mostly based on adult study data and did not look specifically at dysglycemia in adolescents with PCOS.

A few studies have compared HbA1c to OGTT for diagnosing dysglycemia in adolescents with PCOS. One study [5] recommended OGTT independent of the BMI status. Another study [11] supported the use of two-hour glucose vs. fasting glucose values in adolescents with overweight status and obesity. A separate study [12] found that HbA1c had moderate sensitivity and specificity compared with OGTT. Studies involving adult women with PCOS also support better screening modalities and the use of OGTT compared to HbA1c [24, 25], and our findings were consistent with the adult data.

In our study, we analyzed the use of HbA1c as a predictor for IGT. We described the poor predictive ability of HbA1c based on ROC analysis. A normal HbA1c value may be falsely reassuring, and undergoing a formal OGTT may identify dysglycemia in this patient population. Based on the differences we found in patients with and without obesity, we recommend that all patients with a BMI >95th percentile should undergo OGTT. The utility in patients with BMI < 95th percentile and cost effectiveness still needs to be determined.

We also found that higher free testosterone levels correlate with higher insulin resistance (as defined by HOMA-IR). Hence, an elevated free testosterone level may be an indicator of the presence of insulin resistance and may be of additional utility in the assessment of this group of adolescents.

Our study has various limitations. Its retrospective nature did not allow us to assess all patients systematically, and the clinical decision to perform glucose screening was provider dependent. In addition, due to the retrospective design of our study, a comparative non PCOS control group has not been included for comparison. The strengths of our study include assessment of variables aside from glucose tolerance, and a diverse heterogeneous population from a tertiary care urban healthcare center.

Low hepatic sex hormone- binding globulin (SHBG) production may be a key step in the pathogenesis of PCOS. There is emerging evidence that serum SHBG levels may be a useful diagnostic biomarker and therapeutic target for managing women with diagnosis of PCOS [26]. It may be interesting to study the SHBG relationship with obesity in our adolescent patient population as a future direction to our study. In addition, a phenotypical classification of our patient group based on factors such as oligo-anovulation, hyperandrogenism, ethnicity and polycystic ovarian morphology in future may provide further information in subclassification of PCOS phenotype in adolescents with PCOS.

## Conclusions

We have described the presence of glucose abnormalities in adolescent patients diagnosed with PCOS, with increasing rates of IGT and insulin resistance in those with a BMI >95th percentile. We found that HbA1c is a poor predictor of IGT when compared to a standard 75 gram OGTT. In addition, patients with higher free testosterone levels show higher association with insulin resistance irrespective of BMI – an association that requires further study. We believe that all adolescent patients with PCOS and BMI > 95th percentile should undergo a formal OGTT in order to further help identify individuals who may be at risk of developing type 2 diabetes and may potentially benefit from early initiation of medical therapies and diet and lifestyle changes.

## Data Availability

All data generated or analyzed during this study are included in this published article.

## Abbreviations

PCOS: Polycystic Ovary Syndrome
IGT: Impaired Glucose Tolerance
BMI: Body Mass Index
HbA1c: Hemoglobin A1c (HbA1c)
OGTT: Oral Glucose Tolerance Test
HOMA-IR: Homeostasis Model Assessment
WBISI: Whole Body Insulin Sensitivity Index
ROC: Receiver Operating Characteristic
AUC: Area Under the Curve
AES: Androgen Excess Society
ADA: The American Diabetes Association
HPLC: High-Performance Liquid Chromatography
β: Beta estimate
IQR: Interquartile Range
SHBG: Sex Hormone-Binding Globulin
